# Right Atrial Fibroelastoma Presenting as Typical Atrial Flutter: Rare Disease in Unusual Location

**DOI:** 10.1155/2017/4764587

**Published:** 2017-10-10

**Authors:** Ahmad Abuarqoub, Ghada Elshimy, Muhammed Shittu, Aiman Hamdan, Fayez Shamoon

**Affiliations:** Department of Cardiology, Saint Joseph's Regional Medical Center, 703 Main St, Paterson, NJ 07503, USA

## Abstract

Typical atrial flutter as initial presentation of papillary fibroelastoma involving the cavotricuspid isthmus is not described before in literature. To our knowledge only 14 cases have been reported in literature involving the right atrium. Very unusual location is at the junction between inferior vena cava (IVC) and right atria as only 1 case has been reported.

## 1. Case Report

**Figure 1:**
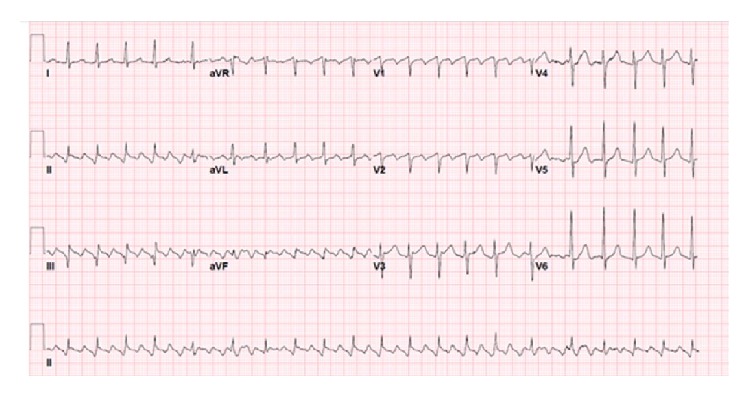
EKG showing typical atrial flutter with variable AV block.

**Figure 2:**
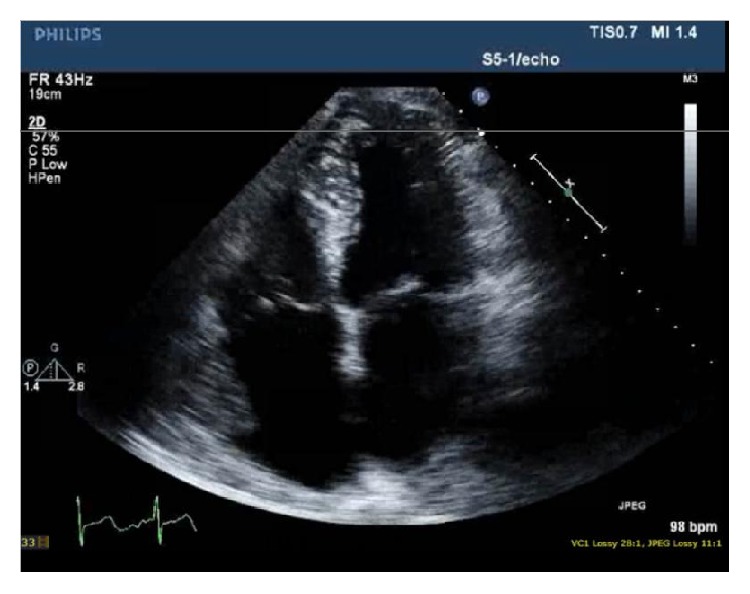
Apical 4 chamber view of 2D echocardiogram showing right atrial mass/clot.

**Figure 3:**
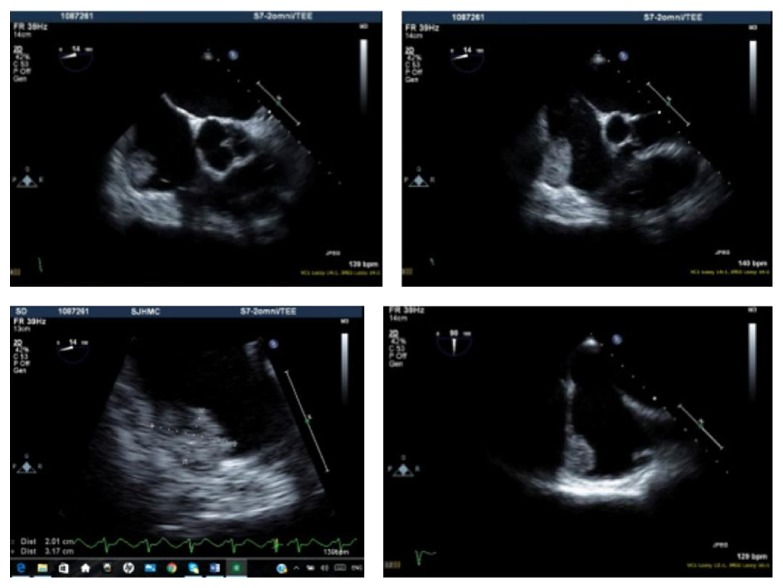
Aortic valve short axis and bicaval views of TEE showing right atrial mass at the junction between the SVC and the right atrium.

**Figure 4:**
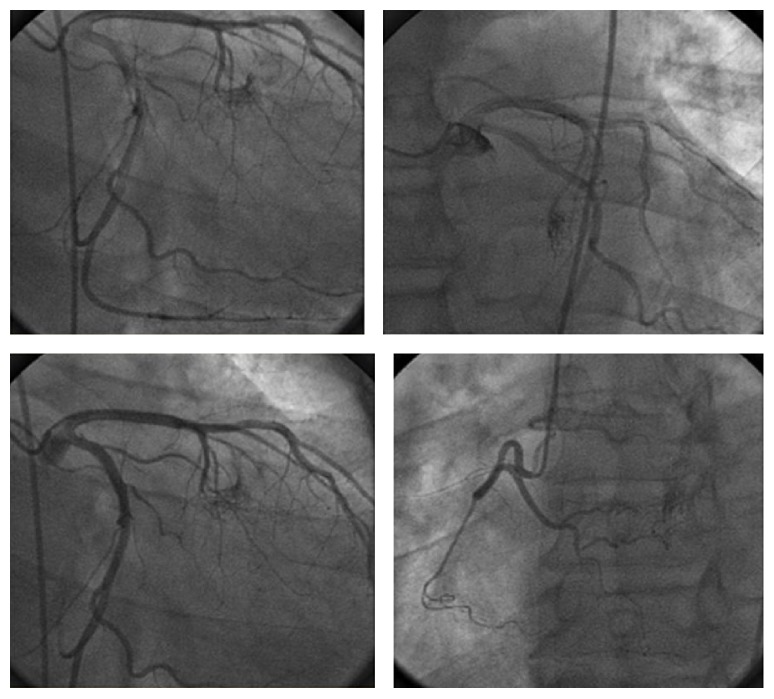
Coronary angiogram showing coronary cameral fistula between the first septal perforator of LAD and LV and between the RCA and LV.

**Figure 5:**
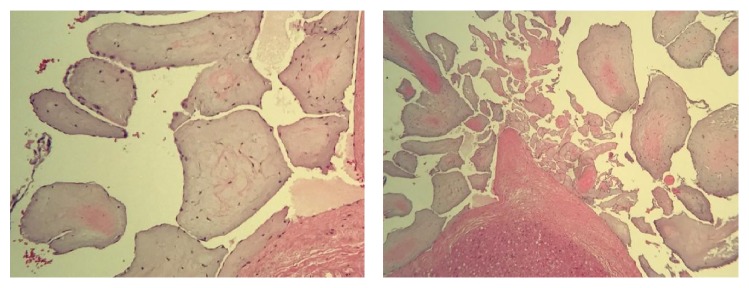
H and E stain of the excised right atrial mass showing fragments of the myocardium and multiple free floating papillary structures. The outer surface of these papillary structures is lined by endothelial cells. The stroma is fibroelastic with prominent myxoid changes. These findings are consistent with papillary fibroelastoma.

59-year-old African American heavy smoking male with past medical history of hypertension, diabetes, and dyslipidemia presented to our facility with sudden onset of palpitations, which were rapid and irregular and started 1 day prior to presentation associated with increased shortness of breath, and denied any history of orthopnea, PND, chest pain, or lower limb edema. EKG was done and showed typical atrial flutter with variable AV block and rapid ventricular response (Figure 1). Transthoracic echocardiography (TTE) showed the left ventricular systolic function 40–45% with right atrial mass possibly a clot extending to right atrial appendage measuring 2 cm × 3.1 cm (Figure 2). Transesophageal echocardiography (TEE) was done for further evaluation of the lesion which showed atrial mass at the conjunction between IVC and right atria (Figure 3). Preoperative cardiac catheterization showed no obstructive coronary artery disease, except for coronary cameral fistula (Figure 4). Surgical excision of the right atrial mass from base of the stalk was done and histopathology of the mass was consistent with PFE (Figure 5). The patient had smooth postoperative course, ablation of the IVC isthmus was not done, hence the atrial arrhythmia was attributed to the compressive effect of the tumor, and the patient maintained sinus rhythm since then. The patient was discharged on anticoagulation; 6-month follow-up transthoracic echocardiogram was done and showed no residual mass.

## 2. Discussion

Papillary fibroelastoma (PFE) is the third-most common benign cardiac tumor followed by atrial myxoma and lipoma [1]. They are small, avascular tumors composed of a dense core of connective tissue, an intermediate layer of loose connective tissue, and a superficial layer of hyperplastic endothelial cells [2], usually located on the mitral or aortic valvular endocardium (less common on tricuspid and pulmonary valvular endocardium) [3]. Origin from the right atrium is rarely seen, often misdiagnosed as atrial myxoma or thrombus. Cardiac PFEs account for 7% of all cardiac tumors. They are rarely found along the atrial or ventricular walls [4].

The pathophysiology of PFEs is controversial. They have been considered to be neoplasms, hamartomas, organized thrombi, and endocardial responses to infection or hemodynamic turbulence leading to endothelial hyperplasia [4].

Papillary fibroelastomas are of clinical relevance because of their potential for embolization leading to cardiovascular and neurological embolic-like symptoms when arising from the left side of the heart [1, 2, 5]. However, right-sided cardiac tumors remain predominantly asymptomatic until they become large enough to interfere with intracardiac blood flow, alter hemodynamic function, or induce arrhythmias. In addition, paradoxical emboli can occur in the setting of a right to left shunt [2, 5]. This type of mass can be discovered most precisely by TEE as it has sensitivity of 88.9% and specificity of 87.8%. TEE is more sensitive than TTE if tumor size is less than 20 mm. Other diagnostic modalities are computed tomography and gadolinium enhanced magnetic resonance imaging [2, 6, 7].

Surgical removal of these tumors is debatable. The decision to operate is mainly dependent on the size, location, and mobility of the mass. However, it is almost unanimously agreed upon that surgery is necessary once cardiovascular or neurological symptoms occur. Prior to surgery, the use of prophylactic anticoagulants decreased the risk of systemic embolization [4].

Hakemi et al. reviewed 13 reported cases of nonvalvular right atrial PFE and most of these cases were diagnosed incidentally during routine work-up. Only one additional case was reported since then leaving us with a total of 14 cases of right atrial PFE [8, 9].

The unique features of our case are its unusual location at the junction between IVC and right atrium and presenting as typical atrial flutter in regard to its location. The presence of coronary cameral fistula is an incidental finding as it has no association with PFE in literature review. In the setting of large right atrial mass and the presence of atrial arrhythmia surgery was considered. Anticoagulation was given in the setting of CHADS2Vasc score of 2, keeping in mind that the patient did not have any systemic embolic manifestations from the right atrial mass. Repeat echocardiogram did not show any residual mass.

## 3. Conclusion

Right atrial cardiac papillary fibroelastoma is an extremely rare finding. Only 14 cases have been previously reported in the literature. This is the first report of such a tumor in the junction between right atrium and IVC. Surgical removal of these tumors is a point of debate. However, surgery is recommended in large and symptomatic masses. Anticoagulation is required in patients with systemic embolic manifestations.

## Abbreviations

PFE: Papillary fibroelastoma
IVC: Inferior vena cava
SVC: Superior vena cava
TTE: Transthoracic echocardiography
TEE: Transesophageal echocardiography.
